# New section in *EJNMMI* and *Annals of Nuclear Medicine*

**DOI:** 10.1007/s12149-016-1125-4

**Published:** 2016-09-16

**Authors:** Ignasi Carrió, Seigo Kinuya

**Affiliations:** 1 Department of Nuclear Medicine, Autonomous University of Barcelona, Barcelona, Spain; 2 Department of Nuclear Medicine, Kanazawa University Graduate School of Medical Science, Kanazawa, Japan

Dear Readers,

The *EJNMMI* and *Annals of Nuclear Medicine* are introducing a new section that brings a review of the relevant articles published last year in the respective journals. Written by guest key experts, this section is intended to offer the readers a condensed global view on the high-quality research work that has been published in the other journal during the last period. We hope that this new journal feature will strengthen scientific cooperation between Europe and Japan and will be interesting and informative to the entire readership.

Ignasi Carrió, *EJNMMI* Editor-in-Chief

Seigo Kinuya, *Annals of Nuclear Medicine* Editor-in-Chief

